# Supplementary material for: French validation of the Weight Efficacy Life-Style questionnaire (WEL): Links with mood, self-esteem and stress among the general population and a clinical sample of individuals with overweight and obesity
Source: PLoS One. 2021 Nov 16;16(11):e0259885. doi: 10.1371/journal.pone.0259885 (PMC8594800; doi:10.1371/journal.pone.0259885)
Supplement: Attachment — Submitted filename: Response to Reviewers.docx [file pone.0259885.s001.docx]

Manuscript PONE-D-21-08991

**Response to Reviewers**

Dear Dr. Palazón-Bru,

Thank you for giving us the opportunity to submit a revised draft of the manuscript “French validation of the Weight Efficacy Life-Style questionnaire (WEL): Links with mood, self-esteem and stress among the general population and a clinical sample of individuals with overweight and obesity” for publication in the PLOS ONE journal.

We appreciate the time and effort that you and the reviewer dedicated to providing feedback on our manuscript and are grateful for the insightful comments on and valuable improvements to our paper.

We have incorporated all the suggestions made by the reviewer. Those changes are highlighted in red within the document named “Revised Manuscript with track Changes”.

Please see below, in blue, for a point-by-point response to the reviewers’ comments and concerns. All line numbers refer to the revised manuscript file with tracked changes.

Sincerely,

Natalija Plasonja
PhD student at the University of Bordeaux, France
natalija.plasonja@u-bordeaux.fr

**Journal Requirements:**

When submitting your revision, we need you to address these additional requirements.

1. Please ensure that your manuscript meets PLOS ONE's style requirements, including those for file naming. The PLOS ONE style templates can be found at 
https://journals.plos.org/plosone/s/file?id=wjVg/PLOSOne_formatting_sample_main_body.pdf and 
https://journals.plos.org/plosone/s/file?id=ba62/PLOSOne_formatting_sample_title_authors_affiliations.pdf

**Response:** The style requirements of the manuscript have been verified.

2. Please review your reference list to ensure that it is complete and correct. If you have cited papers that have been retracted, please include the rationale for doing so in the manuscript text, or remove these references and replace them with relevant current references. Any changes to the reference list should be mentioned in the rebuttal letter that accompanies your revised manuscript. If you need to cite a retracted article, indicate the article’s retracted status in the References list and also include a citation and full reference for the retraction notice.

**Response:** Thank you for pointing this out. All the references were converted to the “Vancouver” style.

3. Please include additional information regarding the survey or questionnaire used in the study and ensure that you have provided sufficient details that others could replicate the analyses. For instance, if you developed a questionnaire as part of this study and it is not under a copyright more restrictive than CC-BY, please include a copy, in both the original language and English, as Supporting Information.

4. Please provide additional details regarding participant consent. In the ethics statement in the Methods and online submission information, please ensure that you have specified (1) whether consent was informed and (2) what type you obtained (for instance, written or verbal, and if verbal, how it was documented and witnessed). If your study included minors, state whether you obtained consent from parents or guardians. If the need for consent was waived by the ethics committee, please include this information.

**Response:** Thank you for the reminder. Information regarding the informed consent of patients and their legal representative are included in the *Methods* section, 2.1.1. ESTEAM cohort (lines 173-180).

If you are reporting a retrospective study of medical records or archived samples, please ensure that you have discussed whether all data were fully anonymized before you accessed them and/or whether the IRB or ethics committee waived the requirement for informed consent. If patients provided informed written consent to have data from their medical records used in research, please include this information.
Once you have amended this/these statement(s) in the Methods section of the manuscript, please add the same text to the “Ethics Statement” field of the submission form (via “Edit Submission”).

For additional information about PLOS ONE ethical requirements for human subjects research, please refer to <http://journals.plos.org/plosone/s/submission-guidelines#loc-human-subjects-research>

**Response:** Thank you for pointing this out. Information regarding the anonymization of the data have been integrated in the Methods section, 2.1.1. ESTEAM cohort (lines 173-180).

5. Thank you for stating the following in the Acknowledgments Section of your manuscript:

The authors would like to give special thanks to Michel Dubourdeaux (R&D Innovation Director at PiLeJe), Christophe Guyomar (Chief Digital Officer at PiLeJe) for their administrative and technical support and Claude Blondeau (Head of Scientific Communication at PiLeJe) for editing the manuscript. Thanks are also addressed to Dr Dominique Périn Calvão for the translation of the MDI, Dr Marilou Bruchon-Schweitzer, Dr Eve Villemur and Dr Gérard Ostermann for their contribution to the translation of the WEL and SSES scales. Anna Brytek-Matera acknowledges financial support by the „Excellence Initiative – Research University” program for years 2020-2026 of the University of Wroclaw.

We note that you have provided funding information that is not currently declared in your Funding Statement. However, funding information should not appear in the Acknowledgments section or other areas of your manuscript. We will only publish funding information present in the Funding Statement section of the online submission form. 
Please remove any funding-related text from the manuscript and let us know how you would like to update your Funding Statement. Currently, your Funding Statement reads as follows:

Anna Brytek-Matera acknowledges financial support by the „Excellence Initiative – Research University” program for years 2020-2026 of the University of Wroclaw.

Please include your amended statements within your cover letter; we will change the online submission form on your behalf.

**Response:** Thank you for pointing this out. The funding statement was removed from the Acknowledgments section and the manuscript and was added to the updated version of the cover letter, which was then completed with additional information regarding the funding of this research.

6. Please include your full ethics statement in the ‘Methods’ section of your manuscript file. In your statement, please include the full name of the IRB or ethics committee who approved or waived your study, as well as whether or not you obtained informed written or verbal consent. If consent was waived for your study, please include this information in your statement as well.

**Response:** The full ethics statement was added in the *Methods* section, 2.1.1. ESTEAM cohort (lines 173-180).

[Note: HTML markup is below. Please do not edit.]

**Review Comments to the Author**

Please use the space provided to explain your answers to the questions above. You may also include additional comments for the author, including concerns about dual publication, research ethics, or publication ethics. (Please upload your review as an attachment if it exceeds 20,000 characters)

Reviewer #1: Dear Dr,
I have gone through the manuscript and feel the manuscript was well written by the authors in this work on French validation of the Weight Efficacy Life-Style questionnaire (WEL): Links with mood, self-esteem and stress among the general population and a clinical sample of individuals with overweight and obesity. However, a minor revision is needed to improve the quality of this paper.

**Response:** Thank you very much.

Abstract: In the design, the authors mentioned multiple statistical analyses. Please report the most important/significant; this will improve the result section, which in the current version is hard to understand where the major benefits happened.

**Response:** Thank you for your comment. Your suggestion was taken into account and the abstract was revised accordingly (lines 31-36).

Introduction:
Please clarify following question into Introduction section:
• What is the main question addressed by the research?

**Response:** Thank you for pointing this out. The main question addressed by the research has been made clearer at the end of the *Introduction* section (lines 142-147).

• How original is the topic? What does it add to the subject area compared with other published material?

**Response:** This suggestion has been taken into account and additional information has been provided (lines 142-147).

• Please show the gap clearly. What is already known and what new knowledge this work will provide?

**Response:** Thank you for your suggestion. Additional information has been included in the *Introduction* section (lines 142-147).

Discussion:

What are the policy implications of these findings?

**Response:** Our findings may feed into the formulation of the national health policy and strategies in obesity prevention and intervention efforts. This idea has briefly been addressed at the beginning of the last paragraph of the *Discussion* (lines 674 – 677).

How can your findings inform improvements in existing nutritional interventions?
What is the barrier to incorporating its use in public health?

**Response:** These two questions have been addressed in the last paragraph of the *Discussion* (lines 674 – 683)*.*

If a public health manager wants to scale up this intervention, would this be feasible?

**Response:** The public health manager needs a multidisciplinary team for creating and implementing the nutritional intervention.
